# The impact of monthly campaigns and other high-profile media coverage on public interest in 13 malignancies: a Google Trends analysis

**DOI:** 10.3332/ecancer.2020.1154

**Published:** 2020-12-10

**Authors:** Samuel A Cohen, Landon E Cohen, Jonathan D Tijerina

**Affiliations:** 1 Stanford University School of Medicine, 291 Campus Drive, Stanford, 94305 CA, USA; 2 Keck School of Medicine of USC, 1975 Zonal Avenue, Los Angeles, 90089 CA, USA; 3 Bascom Palmer Eye Institute, 900 NW 17th Street, Miami, 33136 FL, USA

**Keywords:** Google Trends, cancer, awareness, public interest, media

## Abstract

It is estimated that more than 600,000 people will die of cancer in the United States in 2020. Annual cancer diagnoses throughout the country are expected to rise in the coming years, which may further strain the American healthcare system. As such, it is vital that public health campaigns intended to reduce cancer morbidity and mortality are successful. Monthly cancer awareness campaigns have been used in the past to raise awareness and funding for various malignancies. One notable example is the ‘Pink October’ campaign to raise awareness for breast cancer. There has been limited study, however, on the effectiveness of cancer awareness campaigns for other cancers such as colorectal cancer, prostate cancer and cervical cancer. High-profile media coverage of celebrity cancer diagnoses and/or cancer-related deaths is another method by which knowledge of common cancers is dispersed to the public. In this study, we evaluate the impact of monthly cancer awareness campaigns as well as celebrity cancer diagnoses and/or deaths on Internet search traffic regarding various malignancies.

We used the Google Trends database to evaluate public interest in 13 different cancers (and their respective cancer screening methods, when applicable) from January 2010 to June 2020. Public interest in 6 of 13 cancers (cervical cancer, colorectal cancer, skin cancer, ovarian cancer, breast cancer and lung cancer) was significantly higher in their respective awareness months when compared to the rest of the year. Furthermore, peak public interest for 9 of 13 cancers was associated with a media-event such as a monthly awareness campaign or celebrity diagnoses and/or death. Our findings illustrate the important role that the media plays in facilitating public interest in common cancers and their screening methods. Cancer awareness months can serve as an effective tool to increase Internet search traffic regarding a given malignancy. In the future, public health agencies can attempt to utilise increased search traffic to better educate the public, raise funds and improve enrolment in cancer screening programmes that reduce cancer morbidity and mortality.

## Background/introduction

Cancer is the second leading cause of death in the United States (U.S.), with recent reports estimating more than 600,000 cancer-related deaths in the U.S. in 2020 alone [1]. Cancer diagnoses are projected to increase approximately 45% from 2010 to 2030 due in part to an ageing population and rising obesity rates [2, 3] . There are an abundance of challenges that the anticipated increase in burden on healthcare resources poses to the U.S. healthcare system. As such, it is imperative that in addition to expanding healthcare infrastructure to account for more cancer diagnoses, public health campaigns designed to educate the public about ways to reduce cancer morbidity and mortality must be effective and well-delivered to target demographics [4–6].

One powerful manner in which the healthcare community can raise awareness and educate the public about common cancers is through monthly cancer awareness campaigns as well as media events such as celebrity diagnoses or deaths. Monthly awareness campaigns differ in scope for various types of cancers; efforts to disseminate education about breast cancer, skin cancer, ovarian cancer, prostate cancer and lung cancer are just a few of the many types of dedicated awareness months throughout the calendar year. During these efforts, non-profit organisations from around the country typically join forces to educate the public about a specific type of cancer and to raise funds for further research into the disease. Previous literature has demonstrated the effectiveness of Breast Cancer Awareness Month both domestically and internationally in countries such as Australia, Canada, Brazil, England and Taiwan [7, 8]. However, there has been limited research that quantitatively examines the effectiveness of monthly cancer awareness campaigns in the U.S. for lesser known or less well-publicised cancers such as colorectal cancer, cervical cancer, haematological malignancies and prostate cancer.

High-profile media coverage of celebrity cancer diagnoses and/or cancer-related deaths is another mechanism by which awareness of common cancers is propagated. Actress Angelina Jolie’s 2013 announcement of her intention to undergo prophylactic bilateral mastectomy is one notable example of media coverage that significantly impacted public interest in breast cancer [9]. Furthermore, Katie Couric’s decision to broadcast her colonoscopy live on the *Today Show* in March 2000 similarly led to an uptick in colonoscopy procedures and inquiries about colorectal cancer in the months following her television appearance [10]. Additionally, public interest in cervical cancer in the UK peaked during Jade Goody’s battle with the disease in 2008 and 2009, and led to a measurable increase in cervical cancer screening [11].

Internet search traffic is one way to measure public interest in cancers as a result of monthly cancer awareness campaigns and other media events such as a celebrity cancer diagnosis or death. When searching for health information online, the first place that many turn is the Google search engine, which accounts for nearly 90 percent of global search traffic [12]. As such, public interest in a search topic as measured by Google traffic can serve as a proxy for assessing national public interest in a given search term. Google Trends (GT) is a free, open source tool launched in 2006 that provides customisable analysis of search term volumes entered into the Google search engine [13]. Previously, GT has successfully been used in epidemiological studies examining public health issues ranging from Zika to Lyme disease to vesicular stomatitis to Ebola to cancer screening [14–18]. GT data has also been used to assess changes in public interest in health-related topics in response to widespread media coverage [19, 20]. The purpose of this study is to use GT to evaluate the impact of monthly cancer awareness campaigns and other high-profile media events on public interest in several different malignancies.

## Methods

### GT output

GT analyses can be customised based on search term, time period and geographic location. Once a search term is entered into the GT tool and temporal and geographic constraints are selected, GT generates outputs that describe the frequency of searches for a given search term relative to maximum popularity within the defined time period. The data are reported as relative search volume (RSV) values, which are computed as the ratio between searches for a given topic and the total amount of Google queries. RSV is reported on a scale of 0–100, with 100 indicating the highest percentage of searches for a given topic relative to all Google queries. A larger RSV indicates a higher proportion of queries for a given search term, and an RSV of 0 at a given time point indicates that at the specified time point, the proportion of queries for the search term was less than 1% of its peak RSV (RSV 100). Using RSV rather than absolute count of Google searches allows for comparison of search volume in geographic regions that have different population densities. If RSV was not normalised, larger states such as Texas, New York and California would typically register the highest search volumes over a given time period. For this study, we used GT’s customisable filters to include searches within the U.S. from January 2010 to June 2020 for all selected terms.

### Search term selection

We used GT to examine public interest in 13 different types of cancers with dedicated awareness months as compiled by the American Cancer Society [21]. Databases of search volumes over time were collected for the following search terms: [‘Cervical Cancer’], [‘Colorectal Cancer’], [‘Testicular Cancer’], [‘Oesophageal Cancer’], [‘Brain Cancer’], [‘Skin Cancer’], [‘Ovarian Cancer’], [‘Prostate Cancer’], [‘Blood Cancer’], [‘Thyroid Cancer’], [‘Breast Cancer’], [‘Lung Cancer’] and [‘Pancreatic Cancer’]. In addition, databases of search volumes for search terms related to screening procedures for three cancers (cervical cancer, colorectal cancer and breast cancer) were also measured to determine the effect of awareness campaigns on public interest in popular cancer screenings. The search terms [‘Pap Smear’] and [‘Pap Test’] were selected as the screening search terms related to cervical cancer. The search term [‘Colonoscopy’] was selected as the screening search term related to colorectal cancer. The search term [‘Mammogram’] was selected as the screening search term related to breast cancer.

### Statistical analysis

After databases of search volumes were collected for all search terms included in the study, mean RSV from each cancer’s awareness month was compared to mean RSV for the rest of the calendar year using one-tailed two-independent sample *t*-tests. For example, for a cancer with an awareness month in January, January mean RSV for the given cancer from 2010–2020 was compared to mean RSV for all other months of the calendar year (February–December) over the same time period. One-tailed rather than two-tailed tests were utilised because our hypothesis proposes that mean RSV in a respective cancer’s awareness month will be greater than mean RSV in other months of the year. For screening search terms, mean RSV from the cancer awareness month associated with the respective screening measure was compared to mean RSV for the rest of the year. For example, for the search term [‘Mammogram’] mean RSV from October (Breast Cancer Awareness Month) was compared to mean RSV for all other months. Additionally, in order to determine which cancers are most widely searched on Google, GT was used to compare RSV for the 5 most common cancers in the U.S.: breast cancer, lung cancer, prostate cancer, colorectal cancer and skin cancer [22].

Finally, the peak RSV (100) for each type of cancer was noted. After the peak was identified, an Internet search was used based on the date of the respective peak to determine if public interest was sparked by a media or celebrity event.

All statistical and trend analyses were performed using Microsoft Excel Version 15.21.1 and SPSS Version 26.0.0.1. We defined statistical significance at *p* < 0.05.

## Results

### Awareness month versus non-awareness months

Of the 13 cancers selected for inclusion in this study, public interest in 6 cancers was significantly greater in the given cancer’s indicated awareness month compared to the rest of the calendar year. Public interest in [‘Cervical Cancer’] (*p* = 0.013), [‘Colorectal Cancer’] (*p* < 0.001), [‘Skin Cancer’] (*p* < 0.001), [‘Ovarian Cancer’] (*p* < 0.001), [‘Breast Cancer’] (*p* < 0.001) and [‘Lung Cancer’] (*p* = 0.035) was greater in the months of January, March, May, September, October and November, respectively. Public interest in designated awareness months for [‘Pancreatic Cancer’], [‘Prostate Cancer’], [‘Blood Cancer’], [‘Testicular Cancer’], [‘Oesophageal Cancer’], [‘Brain Cancer’] and [‘Thyroid Cancer’] was greater than throughout the rest of the calendar year; however, this difference was not significant (Table 1).

For the search terms related to cancer screening, public interest in [‘Mammogram’] was significantly greater during Breast Cancer Awareness Month (October) than the rest of the calendar year. Public interest in [‘Pap Smear’], [‘Pap Test’] and [‘Colonoscopy’] was higher in their respective awareness months than throughout the rest of the calendar year; however, this difference was not significant (Table 2).

### Visuals tracking public interest for common cancers

Visuals tracking public interest over time for the cancers included in this study from January 2010–June 2020 are shown below. Data points coloured red represent the awareness month in each calendar year. The first six visuals depict public interest in the cancers which demonstrated greater public interest in their awareness months than throughout the rest of the calendar year, as well as brief descriptions describing whether or not peak RSV corresponded to a media event.

### Cervical cancer

**Figure 1.**
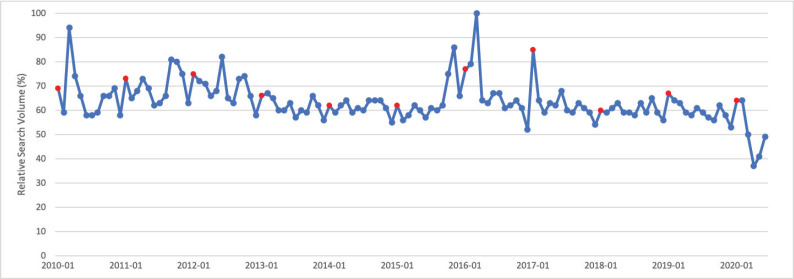
Cervical cancer RSV January 2010–June 2020 [Source: Google Trends, 2020].

January is Cervical Cancer Awareness Month. Public interest in cervical cancer in January was significantly greater than public interest in cervical cancer throughout the rest of the calendar year (*p* = 0.013). Peak interest in cervical cancer was in March 2016, which does not appear to correspond with a media event (Figure 1).

### Colorectal cancer

**Figure 2.**
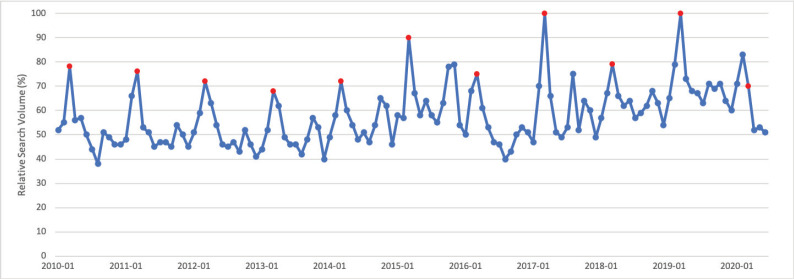
Colorectal cancer RSV January 2010–June 2020 [Source: Google Trends, 2020].

March is Colorectal Cancer Awareness Month. Public interest in colorectal cancer in March was significantly greater than public interest in colorectal cancer throughout the rest of the calendar year (*p* < 0.001). For 10 of 11 calendar years studied, March had the highest relative search volume compared with all other months. Peak interest in colorectal cancer was in March 2017, which corresponds to Colorectal Cancer Awareness Month (Figure 2).

### Skin cancer

**Figure 3.**
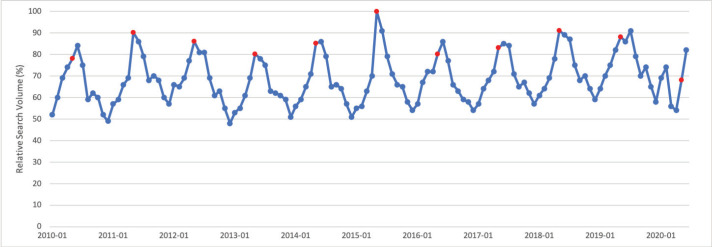
Skin cancer RSV January 2010–June 2020 [Source: Google Trends, 2020].

May is Skin Cancer Awareness Month. Public interest in skin cancer in May was significantly greater than public interest in skin cancer throughout the rest of the calendar year (*p* < 0.001). Public interest in skin cancer showed a seasonal trend, with greater search interest in the summer months and less interest in the winter months. Peak interest was observed in May 2015, which corresponds to Skin Cancer Awareness Month (Figure 3).

### Ovarian cancer

**Figure 4.**
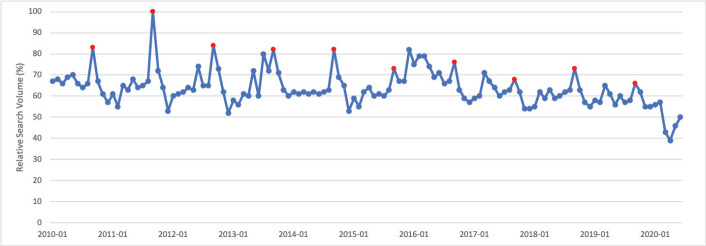
Ovarian cancer RSV January 2010–June 2020 [Source: Google Trends, 2020].

September is Ovarian Cancer Awareness Month. Public interest in ovarian cancer in September was significantly greater than public interest in ovarian cancer throughout the rest of the calendar year (*p* < 0.001). Peak interest in ovarian cancer was observed in September 2011, which corresponds to Ovarian Cancer Awareness Month (Figure 4).

### Breast cancer

**Figure 5.**
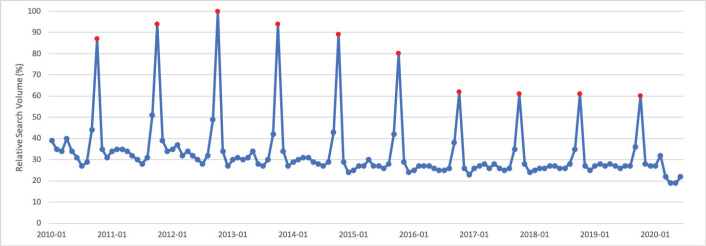
Breast cancer RSV January 2010–June 2020 [Source: Google Trends, 2020].

October is Breast Cancer Awareness Month. Public interest in breast cancer in October was significantly greater than public interest in breast cancer throughout the rest of the calendar year (*p* < 0.001). For all 10 calendar years studied, October had the highest relative search volume compared with all other months. Peak interest in breast cancer was observed in October 2012, which corresponds to Breast Cancer Awareness Month (Figure 5).

### Lung cancer

**Figure 6.**
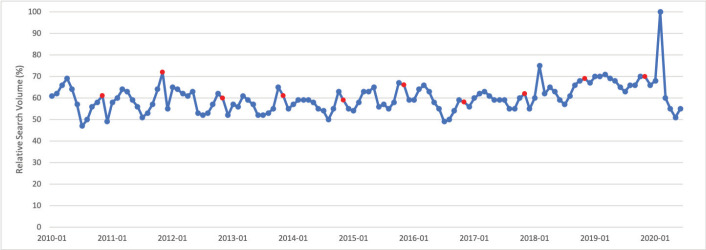
Lung cancer RSV January 2010–June 2020 [Source: Google Trends, 2020].

November is Lung Cancer Awareness Month. Public interest in lung cancer in November was significantly greater than public interest in lung cancer throughout the rest of the calendar year (*p* = 0.035). Peak interest in lung cancer was in February 2020, which corresponds to the month that Rush Limbaugh announced on his radio show that he was diagnosed with lung cancer (Figure 6).

Public interest in oesophageal cancer, brain cancer and pancreatic cancer was not significantly greater during their respective awareness months than their non-awareness months; however, peak RSV (100) for all three cancers was associated with media events, and visuals displaying public interest for all three cancers are shown below.

### Oesophageal cancer

**Figure 7.**
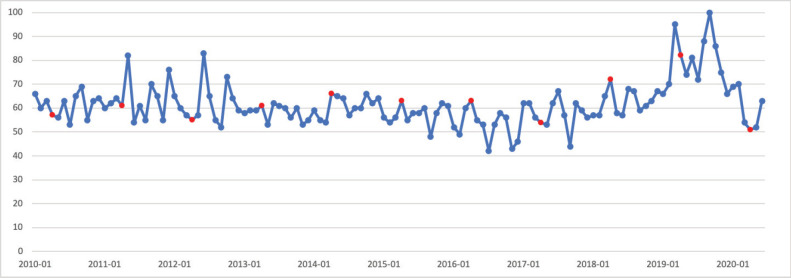
Oesophageal cancer RSV January 2010–June 2020 [Source: Google Trends, 2020].

April is Oesophageal Cancer Awareness Month. Public interest in oesophageal cancer in April was not significantly greater than public interest in oesophageal cancer throughout the rest of the calendar year (*p* = 0.389). Peak interest in oesophageal cancer was in September 2019, the month that former singer Eddie Money died of oesophageal cancer (Figure 7).

### Brain cancer

**Figure 8.**
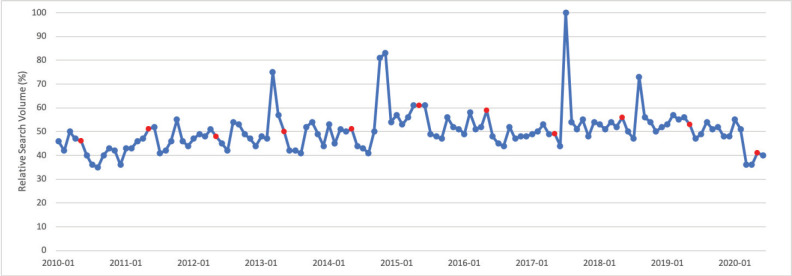
Brain cancer RSV January 2010–June 2020 [Source: Google Trends, 2020].

May is Brain Cancer Awareness Month. Public interest in brain cancer in May was not significantly greater than public interest in brain cancer throughout the rest of the calendar year (*p* = 0.320). Peak interest in brain cancer was in July 2017, the month that former presidential candidate and senator John McCain was diagnosed with a brain tumour (Figure 8).

### Pancreatic cancer

**Figure 9.**
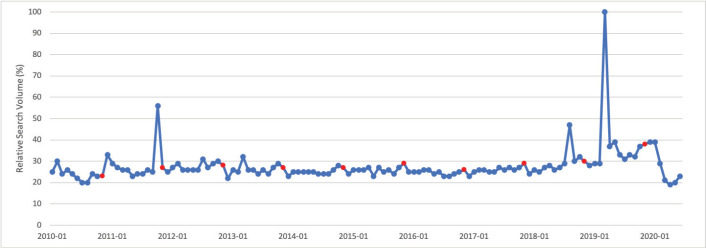
Pancreatic cancer RSV January 2010–June 2020 [Source: Google Trends, 2020].

November is Pancreatic Cancer Awareness Month. Public interest in pancreatic cancer in November was not significantly greater than public interest in pancreatic cancer throughout the rest of the calendar year (*p* = 0.373). Peak interest in pancreatic cancer was in March 2019, which corresponds to the month that Alex Trebek announced to Jeopardy! viewers that he was diagnosed with pancreatic cancer (Figure 9).

Public interest in testicular cancer, prostate cancer, blood cancer and thyroid cancer was not significantly greater during their respective awareness months than in non-awareness months and, to the best of our knowledge, their peak RSV was not associated with a media event. As such, visual outputs displaying public interest for those four cancers were not further analysed.

### Relative public interest in five most common cancers

**Figure 10.**
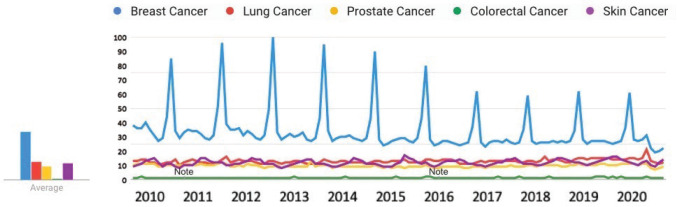
Public interest in breast cancer, lung cancer, prostate cancer, colorectal cancer and skin cancer, January 2010–June 2020 [Source: Google Trends, 2020].

From January 2010 to June 2020, public interest in breast cancer was the highest, followed by lung cancer, skin cancer, prostate cancer and colorectal cancer (Figure 10).

## Discussion

The purpose of this study was to evaluate the effectiveness of cancer awareness campaigns and the effects of other media and celebrity events on public interest in several common types of cancers. Our analysis revealed that from January 2010–June 2020, [‘Cervical Cancer’], [‘Colorectal Cancer’], [‘Skin Cancer’], [‘Ovarian Cancer’], [‘Breast Cancer’] and [‘Lung Cancer’] all had significantly greater public interest as indicated by Google search volumes in their respective awareness months than during the rest of the calendar year. Furthermore, highest levels of public interest in [‘Oesophageal Cancer’], [‘Pancreatic Cancer’] and [‘Lung Cancer’] were not associated with awareness months but rather corresponded to high-profile media events such as a celebrity diagnosis announcement or a celebrity death. With regard to public interest in search terms associated with cancer screening, only [‘Mammogram’] saw a significant increase in public interest during its corresponding awareness month. Public interest in [‘Pap Smear’], [‘Pap Test’] and [‘Colonoscopy’] did not increase during their corresponding awareness months. To the best of our knowledge, our study is the first to quantitatively measure the success of monthly awareness campaigns with GT data for such a wide array of cancers and their associated screening methods. Our results indicate that the media can play a significant role in facilitating public interest in several types of cancers and their corresponding screening methods. Cancer awareness months can serve as an effective tool to increase Internet search traffic regarding a given malignancy. Public health agencies can attempt to utilise increased search traffic to better educate the public about cancer screening methods, raise funds and improve enrolment in cancer screening programmes that reduce cancer morbidity and mortality.

The general purpose of a cancer awareness campaign is to increase awareness regarding a specific malignancy, and raise funds for research into its cause, prevention, detection, treatment and cure [8]. Our results indicate that monthly cancer awareness campaigns for [‘Cervical Cancer’], [‘Colorectal Cancer’], [‘Skin Cancer’], [‘Ovarian Cancer’], [‘Breast Cancer’] and [‘Lung Cancer’] were successful in increasing public interest during monthly awareness campaigns compared with the rest of the calendar year. Our results align with previous research that describes a comparable increase in Google search traffic related to breast cancer during Breast Cancer Awareness Month [7, 23]. However, our findings indicate that monthly awareness campaigns for [‘Cervical Cancer’], [‘Colorectal Cancer’], [‘Skin Cancer’], [‘Ovarian Cancer’] and [‘Lung Cancer’] have also successfully increased Google search traffic, which is contrary to a previous report that found little evidence to support the effectiveness of non-breast cancer monthly awareness campaigns [24]. In contrast to the methods used in the referenced paper, our study used statistical analyses such as two-sample independent *t*-tests to compare GT data in awareness months versus non-awareness months, while the previous study solely identified sharp peaks in RSV from baseline and did not use any statistical tests to evaluate the relationship between monthly cancer awareness campaigns and corresponding GT search traffic.

Additionally, our findings align with previous research indicating that celebrity announcements can significantly increase public interest in health-related topics. Peak public interest in [‘Oesophageal Cancer’], [‘Pancreatic Cancer’] and [‘Lung Cancer’] was all related to either a celebrity diagnosis or a celebrity death. Tijerina *et al* [19] reported that celebrity announcements by Angelina Jolie, Kylie Jenner and Caitlyn Jenner resulted in greatly increased Google search volumes about their respective procedures following their public announcements about prophylactic mastectomy, lip augmentation and gender affirming surgery. Our results suggest that in addition to planned awareness campaigns, spontaneous celebrity announcements often increase Google traffic related to various cancers and may provide an excellent opportunity to both fundraise and provide awareness about screening protocols for the cancer of interest. What’s more, previous research shows that this surge in Google traffic following celebrity announcements can eventually result in health behaviour change. Following Angelina Jolie’s announcement about her decision to undergo a prophylactic mastectomy in 2013, there was a 285% increase in breast and ovarian cancer referrals as well as an 80% increase in breast cancer gene (BRCA) tests [25]. Additionally, there was an increase in colonoscopies in the months after Katie Couric launched her colorectal cancer awareness campaign on the *Today Show* in March 2000 [10]. With these relationships in mind, organisations charged with implementing cancer awareness campaigns in the future should evaluate the potential use of spontaneous cancer-related celebrity announcements as a springboard mechanism to drive attention and resources to their cause, even if these announcements fall outside of traditional awareness months.

The two most common goals of cancer awareness campaigns are 1) educate the public to increase cancer screenings and improve early detection of cancer, when applicable and 2) raise funds for research into the disease. As it relates to increasing cancer screenings in order to detect cancer at an earlier stage, the screening of asymptomatic individuals is recommended for cervical cancer, breast cancer and colorectal cancer [26]. Our results indicate that despite increased Google search traffic for the aforementioned cancers in their respective awareness months, only the screening search term related to breast cancer ([‘Mammogram’]) was also associated with an increase in Google search traffic in its corresponding awareness month. [‘Pap Smear’], [‘Pap Test’] and [‘Colonoscopy’] did not see corresponding increases in public interest during their respective awareness months. The magnitude of the Breast Cancer Awareness Month campaign compared to Cervical and Colorectal Cancer Awareness Month Campaigns as well as the marketing strategies used during Breast Cancer Awareness Month may partially explain this discrepancy. Breast cancer is more common than both cervical cancer and colorectal cancer [1], and awareness campaigns for breast cancer have been particularly successful as a result of large corporate partnerships. For example, the American Cancer Society and the National Football League have partnered since 2009 with their *Crucial Catch: Screening Saves Lives* campaign to remind women about the importance of receiving a mammogram [27]. Furthermore, the increased number of breast cancer diagnoses compared with other cancers has resulted in many celebrities being diagnosed with breast cancer, recovering from the disease and subsequently becoming important faces of the Breast Cancer Awareness Month campaign, where they have large platforms to highlight that screening for breast cancer with a mammogram may lead to early cancer detection. Robin Roberts, Julia Louis-Dreyfus, Christina Applegate and Jennifer Garner are just a few of the many celebrities who have publicly discussed the importance of mammography, and that may help account for increasing effectiveness of the Breast Cancer Awareness Campaign as it relates to improving Google search traffic related to screening [28, 29]. As a result of its tremendous visibility on television and in popular culture, the mammogram is closely associated with breast cancer and Breast Cancer Awareness Month. Future awareness campaigns for Cervical Cancer Awareness Month and Colorectal Cancer Awareness Month should implement strategies used successfully by Breast Cancer Awareness Month to more closely link their awareness campaigns with proper screening measures.

The second primary goal of a cancer awareness campaign is to raise research funds. A recent report suggests that the breast cancer awareness movement is one of the most successful marketing and awareness campaigns in terms of fundraising, with non-profits raising $460 million in 2018 [30]. Lung cancer and colorectal cancer, despite a higher mortality rate than breast cancer [22], only accounted for $92 million and $18 million, respectively, in total annual revenue in 2018 [30]. Our results indicate that public interest in lung cancer and colorectal cancer was greater in their respective awareness months than throughout the rest of the calendar year; however, previous reports suggest that this increased public interest has not necessarily translated into greater fundraising dollars [30]. Recent research indicates that cancers that were associated with stigmatised behaviours, such as lung cancer and its association with smoking, or cervical cancer and its association with sex, were often underfunded [30]. However, colorectal cancer was not associated with a stigmatised behaviour yet was still underfunded relative to its burden on society and the healthcare system [30]. In the U.S., non-profits behind Colorectal Cancer Awareness Month as well as other underfunded cancer awareness months should utilise search engine optimisation strategies in order to convert increased Google search traffic during their respective awareness months into fundraising revenue that can be used for both research and for promoting health behaviours such as life-saving cancer screenings.

There are several limitations to our study. First, although the Google search engine is the most widely used in the world [31], it is one of many search engines that people may use to gather information about cancer. GT has no method for tracking interest in cancers on alternate search engines. Next, GT does not provide information about the demographics of the users whose data are reflected in this study. Previous research indicates that younger patients are more likely to use the Internet as a source of health information [32]. We cannot be certain that the Google health-seeking information reflected in this study is representative of the U.S. population as a whole. Recall bias is another limitation of our study. When determining whether or not peak RSV for a cancer was associated with a media event, a retrospective search was used in an attempt to correlate an announcement with a date range. As such, it is possible that an incorrect association between a media event and GT data was made. However, the consistency with which peak RSV for a given cancer was associated with either an awareness month campaign or a media event appears to support our conclusions. Despite these limitations, we believe that the GT dataset provides valuable information regarding the effect of monthly cancer awareness campaigns on public interest in various malignancies, which can help to shape the direction of future campaigns with the ultimate goal of educating the public and reducing cancer mortality rates in the U.S.

## Conclusions

In conclusion, our results indicate increased Internet search traffic regarding cervical cancer, colorectal cancer, skin cancer, ovarian cancer, breast cancer and lung cancer during their respective cancer awareness months. In the future, public health agencies may utilise this information when devising innovative cancer awareness campaigns that aim to educate the public, raise money for cancer research and improve participation in screening programmes that lead to early cancer detection. Furthermore, outside of traditional monthly awareness campaigns, celebrity diagnoses or deaths associated with a particular cancer may provide an excellent opportunity to further inform the public about the cancer as well as to direct resources and attention to the cause. Our results also demonstrate reduced interest in several cancers such as prostate cancer, haematologic malignancy and thyroid cancer that are less prominent in the public zeitgeist, which may indicate that newer marketing techniques may be needed to effectively target at risk populations.

## Financial disclosure and conflicts of interest

No authors have financial disclosures or conflicts of interest to declare. No funding was received for this article.

## Figures and Tables

**Table 1.** Mean RSV in cancer awareness month versus rest of calendar year for common cancers, 2010–2020.

Search term	Awareness month	Awareness month mean RSV	Non-awareness month mean RSV	*p*
Cervical cancer	January	69.1	63.2	**0.013**
Colorectal cancer	March	80.0	55.7	**<0.001**
Testicular cancer	April	77.2	72.8	0.118
Oesophageal cancer	April	62.3	61.4	0.389
Brain cancer	May	51.4	50.1	0.320
Skin cancer	May	84.5	67.1	**<0.001**
Ovarian cancer	September	78.7	62.4	**<0.001**
Prostate cancer	September	78.5	77.2	0.306
Blood cancer	September	68.6	67.8	0.423
Thyroid cancer	September	70.7	70.2	0.438
Breast cancer	October	78.8	29.8	**<0.001**
Lung cancer	November	63.8	59.8	**0.035**
Pancreatic cancer	November	28.4	27.5	.373

**Table 2.** Mean RSV in cancer awareness month versus rest of calendar year for common screening methods, 2010–2020.

Search term	Awareness month	Awareness month mean RSV	Non-awareness month mean RSV	*p*
Pap smear	January	86.0	82.7	0.100
Pap test	January	74.7	74.5	0.470
Colonoscopy	March	68.5	66.7	0.322
Mammogram	October	69.4	47.5	**<0.001**
